# Activity of wild Japanese macaques in Yakushima revealed by camera trapping: Patterns with respect to season, daily period and rainfall

**DOI:** 10.1371/journal.pone.0190631

**Published:** 2018-01-02

**Authors:** Goro Hanya, Yosuke Otani, Shun Hongo, Takeaki Honda, Hiroki Okamura, Yuma Higo

**Affiliations:** 1 Primate Research Institute, Kyoto University, Inuyama, Japan; 2 Institute for Academic Initiatives, Osaka University, Suita, Japan; 3 Graduate School of Bioagricultural Sciences, Nagoya University, Nagoya, Japan; University of Sydney, AUSTRALIA

## Abstract

Animals are subject to various scales of temporal environmental fluctuations, among which daily and seasonal variations are two of the most widespread and significant ones. Many biotic and abiotic factors change temporally, and climatic factors are particularly important because they directly affect the cost of thermoregulation. The purpose of the present study was to determine the activity patterns of wild Japanese macaques (*Macaca fuscata*) with a special emphasis on the effect of thermal conditions. We set 30 camera traps in the coniferous forest of Yakushima and monitored them for a total of 8658 camera-days between July 2014 and July 2015. Over the one-year period, temperature had a positive effect, and rainfall had a negative effect on the activity of macaques during the day. Capture rate was significantly higher during the time period of one hour after sunrise and during midday. During winter days, macaques concentrated their activity around noon, and activity shifted from the morning toward the afternoon. This could be interpreted as macaques shifting their activity to warmer time periods within a single day. Japanese macaques decreased their activity during the time before sunrise in seasons with lower temperatures. It was beneficial for macaques to be less active during cooler time periods in a cold season. Even small amounts of rainfall negatively affected the activity of Japanese macaques, with capture rates decreasing significantly even when rainfall was only 0.5–1 mm/min. In conclusion, thermal conditions significantly affected the activity of wild Japanese macaques at various time scales.

## Introduction

Organisms have evolved in a world that generally lacks long-term temporal stability; biotic and abiotic influences are subject to variable periodic fluctuations [1, 2]. Molecular clocks generating circadian rhythm are widespread both in animals [3, 4] and plants [5]. This is an adaptation to the rhythmic, cyclic changes of daylight caused ultimately by the rotation of the Earth, and it is related to the animals’ ecological adaptation of using of different time periods in a day [3]. For example, eight mammalian species in Hokkaido, northern Japan, were recognized as diurnal, nocturnal, crepuscular (active at twilight), and cathemeral (active throughout the day) [6]. The visual ability to monitor the environment under various light conditions affects the use of different time periods in a day with different light availability [7]. The utilization of daytime and nighttime is also affected by other abiotic and biotic factors, and this has relevance to feeding, thermoregulation, and anti-predation strategies [8, 9] Moreover, this is one of the important aspects that defines a species’ niche [10].

Circannual molecular clocks are indicated or suggested in many species [11–13], which is an adaptation to the circannual, or seasonal, environmental fluctuations caused ultimately by the revolution of the Earth around the Sun. Seasonal fluctuations include day length, climate, and food availability [1]. As a result, almost all wild animals exhibit seasonal changes in their physiology and behavior, for example hibernation, diet shift and migration [14–20].

The animals’ responses to daily and seasonal fluctuations often need to be analyzed in combination because animals modify their use of various daily time periods in different seasons. For example, crepuscular sika deer increased their activity during daytime in winter [6]. Among cathemeral primates that use both day and night, various factors such as temperature, predation, and food availability affect the allocation of diurnal and nocturnal activities [9, 21, 22]. Some of these factors change seasonally, so it is important to examine both seasonal and daily fluctuations in the activity patterns of species.

Among various environmental fluctuations affecting the life of animals, temperature is one of the most important factors. It can affect animals indirectly via food availability or behavior of the predators [23, 24], but it can also directly influence thermoregulation [25]. Endotherms maintain their body temperature by producing heat inside of their body [26]. Heat production is energetically costly, so behavioral thermoregulation, a behavior that minimizes the cost of physiological thermoregulation, is also important. Such behaviors include the selection of a thermally favorable microenvironment [27, 28] and the suppression of heat loss by modifying their own body posture [29]. Changes in activity in response to temporal fluctuations in temperature could be interpreted, in a broad sense, as one such mode of behavioral thermoregulation [30, 31].

In addition to temperature, rainfall is another climatic factor that relates to the thermoregulatory cost. Rainfall can affect the activities of both ectothermic [32, 33] and endothermic animals [34]. If the heat loss that occurs as a result of getting wet is problematic, animals will decrease their activity level when it rains [34]. Alternatively, if drought is of greater concern than heat loss, animals will increase their activity when it rains [27]. Researchers often use a day or a season as a unit of analysis when assessing the effect of rainfall on activity [35–37]. Rainfall is much less cyclical than seasonal or daily changes in sunlight or temperature [38]. Therefore, analyses at various time-scales are necessary to effectively examine the impacts of rainfall on the focal species.

Camera trapping is a method that evaluates animal activity levels with less bias and at a finer time scale than other methods, such as direct observation [39, 40], live trapping [37, 41] and radio tracking [42]. It is also possible to study both the active and inactive time periods of the animals using a single method [43, 44]. Camera trapping is also cost-effective; after positioning the cameras, we can collect data without much additional research effort until the batteries die (i.e., for a few months), memory of the SD cards is full, or the camera is mechanically broken. Therefore, camera trapping is a powerful tool when evaluating temporal variations. It does not allow researchers to analyze behavioral data in detail, as it is impossible to track the animal for a long time. However, camera trapping would provide enough data to assess the effect of environmental temporal fluctuations on animal behavior in a simple way, such as activity level [6].

The purpose of the present study was to determine the activity patterns of wild Japanese macaques (*Macaca fuscata*) at various time scales, and we placed special emphasis on the role of climatic factors (e.g., temperature and rainfall). We examined seasonal variations, daily variations and the immediate effect of rainfall. We examined the hypothesis that macaques modify their activity in response to thermal stress [45]. We predict that (1) macaques decrease their activity when the temperature is low and rainfall is large over a single year, (2) macaques are most active during the warmest time periods in a day, (3) in winter, macaques shift their activity from the cooler mornings to the warmer afternoons, and their activity is concentrated around the warmest time of day (i.e., noon), (4) in winter, macaques decrease their activity at dawn and dusk, when the temperature is low, and (5) rainfall affects the activity of these macaques negatively over a short time period (30 min).

## Methods

This research complied with the Guidelines for Field Research of Non-human Primates of the Primate Research Institute, Kyoto University, and adhered to the legal requirements of Japan.

### Study site

**Fig 1:**
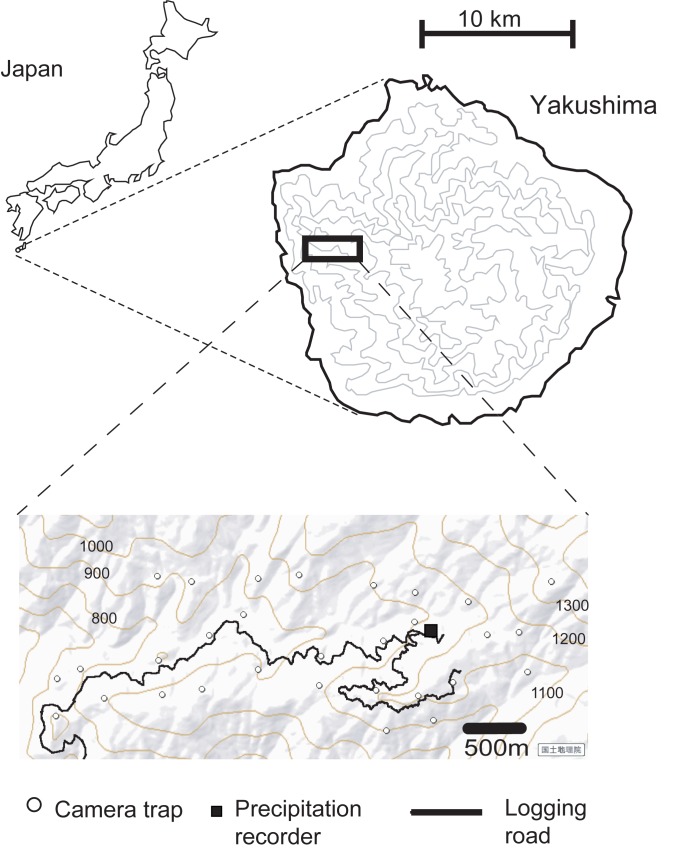
The location of Yakushima, the study area in Yakushima and the camera trapping sites. Contours were drawn every 300 m in the map of Yakushima. The map of the study sites was drawn based on a map provided by the Geospatial Information Authority of Japan.

Yakushima is an island in the southwestern part of Japan (30°N, 131°E), and it encompasses an area of 505 km^2^. We selected a study site in the western part of Yakushima; the site had an area of 7.5 km^2^ and an altitude of 700–1300 m above sea level (Fig 1). The study site included primary forest, naturally regenerated forest that grew after logging in the 1990s, and conifer plantations [46]. The dominant species included warm-temperate evergreen broad-leaved trees, such as *Quercus acuta*, *Q*. *salicina*, *Distylium racemosum*, and conifers, such as *Cryptomeria japonica*, *Abies firma*, and *Tsuga sieboldii*. Small-scale logging was conducted near the logging road. During the census of the macaque population taken in August 2014 and August 2015, we often observed them within close range (<50 m) of the logging site, so we assumed that any immediate effect on capture rate by avoiding the logging site would be small. Logging may potentially affect the overall capture rate by affecting food availability over the long term [46], and it may affect the variations in the capture rate among camera positions. However, spatial variation was out of the scope of this study. No hunting has been conducted in the study site.

### Climate and day length

The Yakushima Forest Ecosystem Conservation Center recorded the precipitation in the study site at an altitude of 1020 m above sea level (Fig 1); records were taken every 10 min for 24 hours and had a precision of 0.5 mm. We used temperature data that were recorded every hour of the day from the Yakushima Meteorological Station, which is located in an eastern coastal village of Yakushima (http://www.data.jma.go.jp/gmd/risk/obsdl/index.php). We calculated the temperature at the site of the precipitation recorder by assuming a temperature lapse rate of 0.6°C/100 m [38]. Data on the timing of sunrise and sunset were collected from the National Astronomical Observatory of Japan (http://eco.mtk.nao.ac.jp/koyomi/).

### Camera trapping

We set 30 camera traps (Trophy Cam HD ® Bushnell, Model119436) in the study site. We originally designed the study site for point censuses of Japanese macaques [46]. The census area was divided into a grid with cell sizes of 500 m × 500 m, and we designated one observation point, typically on a ridge where we could hear the vocalization of macaques well, in each cell. Cameras were set 50 m directly north or south (at opposite sides of the road) at each of the 30 observation points on July 15, 16, and 17, 2014. The distance of 50 m was chosen so that we could set the cameras at locations of various topographies. Before setting the traps, we dismantled the camera and sealed any openings (e.g., around the frame of the sensor) with silicon to prevent moisture from accumulating in the equipment. After closing the cameras, we wrapped the openings with adhesive tape and placed metal covers on them. We then used straps to secure the cameras to tree trunks at a height of 40 cm above the ground. Once the infrared sensor detected animals, the camera took a 30-sec digitized video with 1-sec intervals. We replaced SD cards and batteries (EVOLTA ® Panasonic) in August and December 2014 and in March 2015. We continued camera trapping until June 28-July 5, 2015, after which we collected all the cameras. The initial settings failed on some cameras, which resulted in a lower number of working days than anticipated. None of the cameras experienced any mechanical failures; even when some of them stopped functioning, they resumed after we replaced the SD card or the batteries. No animals, even other species, were filmed in 56% (6626/11801) of the movies, either because the animals moved out of the frame before the camera started filming or because the camera was triggered by a cue other than an animal. These empty movies were excluded from analysis.

### Data analysis

Because of the seasonal variation in day length (10.2–14.2 hours), the actual time each movie was recorded does not provide much information when the data were combined from different seasons. This was particularly the case for investigating the changes of activity around sunrise and sunset. Therefore, we divided the daily time periods into one hour before sunrise (before sunrise, hereafter), one hour after sunrise, midday, one hour before sunset, one hour after sunset, and midnight. We referred to the time between sunrise and sunset as daytime. We distinguished the periods after sunrise and before sunset from other daytime periods to capture detailed changes in activity around sunrise and sunset, which was suggested in a review on primate daily activity that presented data in actual time [47].

To examine the seasonal variation in the capture rates (prediction 1), we constructed a generalized linear mixed model (GLMM). We used detection (filmed/not filmed) on each day for each camera as a response variable, and we used temperature, rainfall on that day and their interaction as fixed factors. To control the possible effect of spatial variation, e.g., distance from the road, we added the camera ID as a random factor. We used the *glmer* function in the *lme4* package of R 3.2.2 to conduct the analysis.

To examine the daily variations in activity, we conducted three kinds of analysis. First, we calculated the expected number of videos that would be taken during the six daily time periods based on the total duration of working camera time in those time periods. We tested whether the capture rate was significantly different among the daily time periods by comparing the observed and expected values using χ^2^ tests with Bonferroni correction (prediction 2).

**Fig 2:**
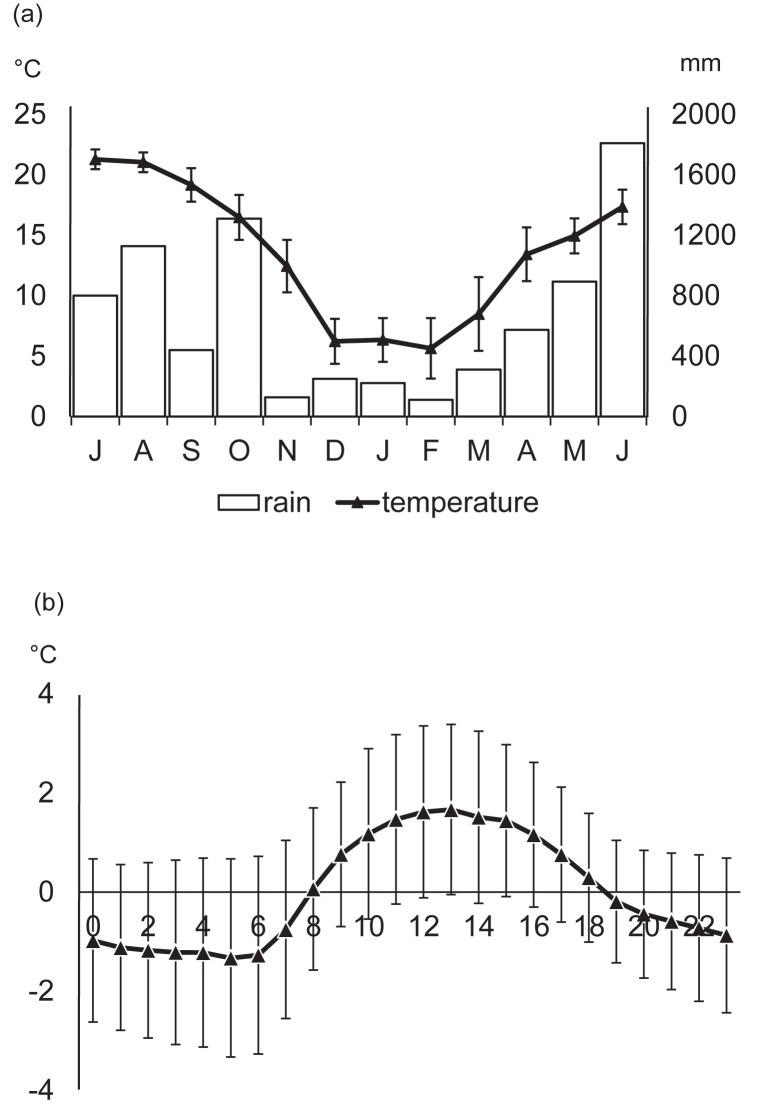
Meteorological record in the study site during the study period. (a) Monthly mean±SD of temperature and rainfall and (b) mean±SD in ambient temperature deviations from the mean temperature on that day. X axis is the time of day.

Second, we calculated the mean and standard deviation (SD) of the times of the filming events and examined whether this varied among seasons (prediction 3). Time-series data are circular in nature, so we calculated circular mean and SD, not the arithmetic values. The average time that the videos were filmed was compared among seasons using the Mardia-Watson-Wheeler test, which is used to test circular data. The seasons were defined as summer (July, August and September), fall (October and November), winter (December, January, February and March), and spring (April, May and June), and they corresponded to the fluctuations in monthly average temperature (Fig 2A). To visualize the daily changes in the activity, we conducted kernel density analysis [48]. We used the *mean*, *sd*.*circular*, *watson*.*wheeler*.*test* and *density*.*circular* functions in the *circular* package of R 3.2.2 for this part of the analysis.

Third, to examine the effect of seasonal changes on the activity during the two transitional time periods (before sunrise and after sunset), we constructed a GLMM. We used the detection of animal(s) by a camera (filmed/not filmed) during a specific period for each day as a response variable, and we used the average daily temperature and the average number of videos taken during the daytime before and after 15 days (in total, 31 days) as fixed factors; finally, the camera ID was used as a random factor. A simple analysis examining only the effect of temperature on the capture rate during the transitional time periods is misleading, since it may only reflect the overall (including midday) increase in capture rate. To avoid this problem, we compared the two models with and without temperature as a fixed factor by the likelihood ratio test (prediction 4). This test revealed whether the temperature can explain the changes in the capture rate during the transitional time period, even after controlling the overall changes in capture rate during daytime [25].

We calculated the total precipitation for a 30-min increment during the daytime. We then tested if the proportion of 30-min times blocks-cameras with filming events changed significantly with given precipitation categories (0.5–1 mm and >1.5 mm) than the frequency without precipitation by χ^2^ tests (prediction 5). To determine the seasonal variation, we divided the data into summer (July, August, and September) and other seasons. This was because the negative impact of heat loss as a result of getting wet might vary with the ambient temperature. We created these precipitation categories to ensure the expected number of videos was greater than five for all categories. We set the alpha level at P<0.05.

## Results

During our study period, the range of ambient temperature was -2.6–28.3°C, and the total precipitation from July 2014 to June 2015 was 7985.5 mm (Fig 2A). The temperature peaked around noon, but the daytime temperature was higher in the afternoon than in the morning (Fig 2B).

**Fig 3:**
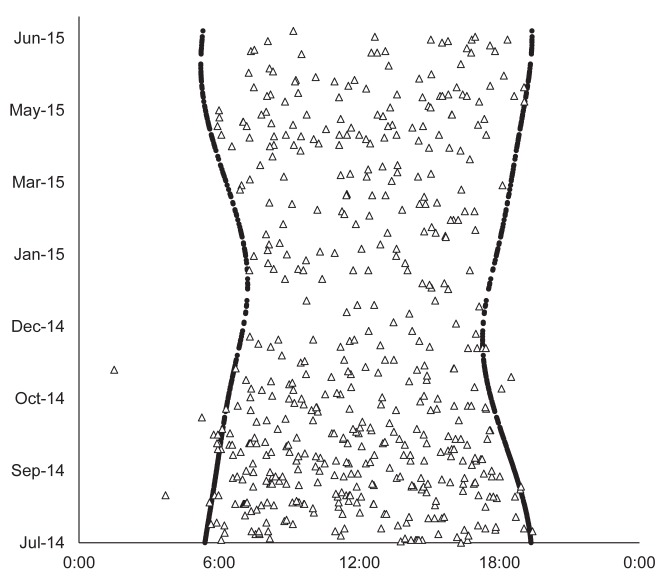
Dates and times when the videos of Japanese macaques were taken (triangle). Circles indicate the time of sunrise and sunset.

We took 631 videos of Japanese macaques, and the capture rate was 0.073/camera/day (Fig 3). The number of working camera-days was 8658 in total and 287±80 days/camera (mean±SD), with a range of 98–352 days. The mean number of daily functioning cameras was 24.8±2.0, with a range of 18–29.

### Seasonal patterns

Over the one year, temperature had a positive effect and rain had a negative effect on the activity of macaques during the day. According to the GLMM analysis, the effects of temperature, rainfall and their interaction on the detection of macaques were all significant (temperature: coefficient = 0.075, P<0.0001; rainfall: coefficient = -0.055, P = 0.0002; interaction: coefficient = 0.0019; P = 0.0014; S4 File). To determine the direction of interaction between temperature and rainfall, we conducted *ad hoc* analysis by dividing the data into two halves of higher and lower temperatures. The rainfall had a more negative effect on activity at lower temperatures (coefficient = -0.031) than at higher temperatures (-0.0087; S4 File).

### Daily patterns

**Fig 4:**
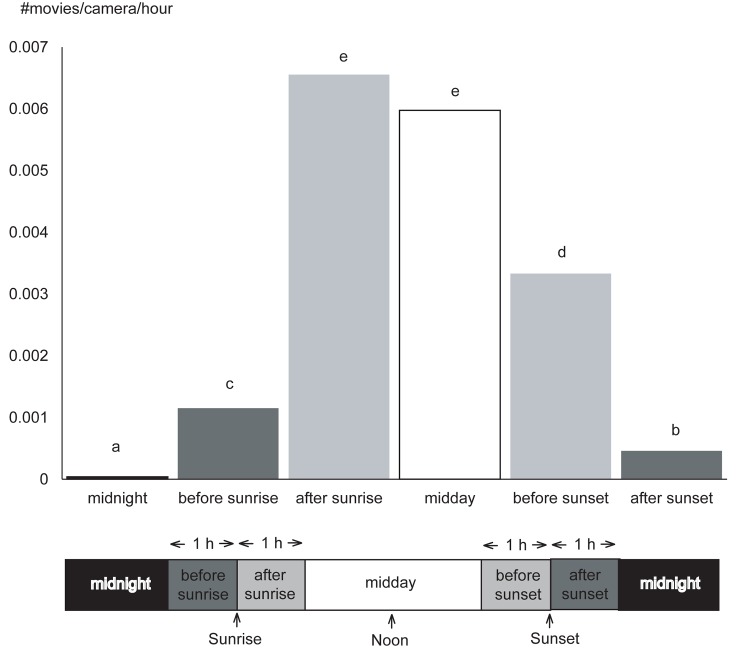
Capture rate of Japanese macaques in the six daily time periods. Significant differences among the time periods (indicated by χ^2^ tests with Bonferroni corrections) are indicated by different lowercase letters in the order a<b<c<d<e.

**Fig 5:**
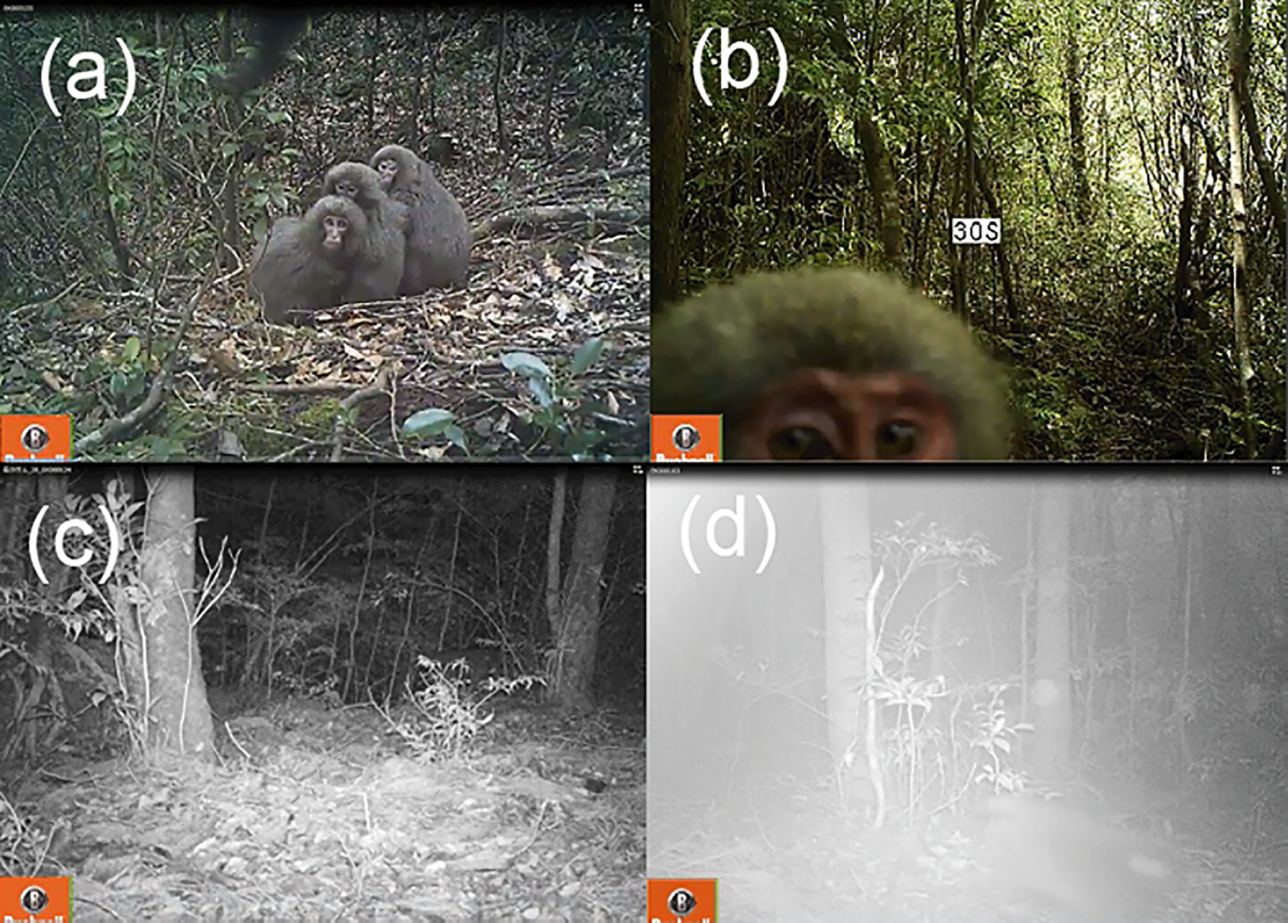
Examples of the movies of Japanese macaques taken by camera trapping in daytime (a and b) and nighttime (c and d). Still images were taken from the movies filmed at (a)17:01 on February 2015, (b)12:01 on August 5, 2014, (c) 1:30 on November 12, 2014, and (d) 18:30 on November 7, 2014.

Capture rates were significantly different among the six daily time periods. The most captures occurred after sunrise and midday, followed by before sunset, before sunrise, and after sunset, and the fewest captures occurred during the midnight period (Fig 4; S4 File). We took three videos during the midnight period (Fig 5): at 3:42 on August 17, at 18:30 on November 7, and at 1:30 on November 12. The animals observed during these times included one adult male and two juveniles of unknown sex. The proportion of activity during the midnight period was 0.5%.

**Fig 6:**
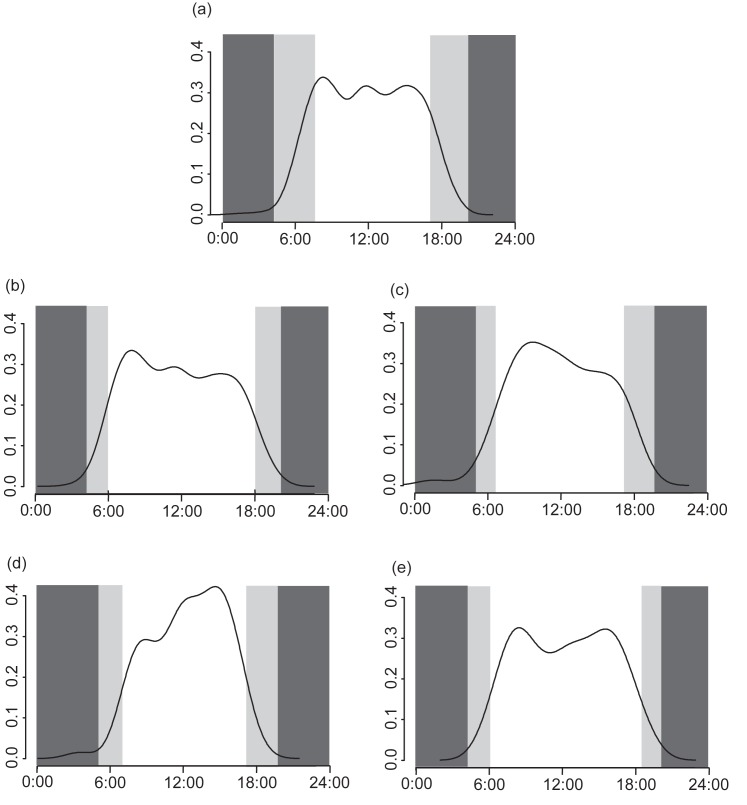
Daily activity patterns of Japanese macaques. Y axis and the curves are the kernel density estimates of the filming events. Light gray areas represent one hour before sunrise and one hour after sunset, and dark gray areas represent nighttime; note that the widths of these areas vary among seasons due to the fluctuating time of sunrise and sunset. (a) All seasons combined, (b) summer (July, August and September), (c) fall (October and November), (d) winter (December, January, February and March), and (e) spring (April, May and June).

During the daytime periods, the average time macaques were filmed changed significantly with the seasons (Fig 6; W = 13.1, df = 6, P = 0.042). The average time was later during the winter (circular average = 12:20) and spring (12:18) than during the fall (11:53) and summer (11:44). The circular standard deviation was the smallest during the winter (3.16 hours), followed by the fall (3.73), spring (3.91) and summer (4.05). To remove the possible effect of different day lengths among seasons, we also calculated the standard deviations during the time periods between 7:14 and 17:18, i.e., times that fell during daytime in all seasons. The standard deviation was still the smallest during the winter (2.92), followed by the fall (3.00), summer (3.12) and spring (3.22).

There was a weak tendency for macaques to be filmed more frequently during the time before sunrise, when the temperature was high, even after controlling for the effect of overall capture rate in the season (S4 File). Likelihood ratio tests indicated that the effect of temperature was only marginally significant during the time before sunrise (χ^2^ = 3.02, P = 0.083) and not significant during the time after sunset (χ^2^ = 2.63, P = 0.104).

### Effect of rainfall

Rainfall, even in small amounts, negatively affected the activity of Japanese macaques. The capture rate during the times with the smallest precipitation (0.5–1 mm/30 min) was significantly lower than the capture rate during the times without precipitation (S4 File). However, when we analyzed the data in summer only, the capture rate during the times with the smallest precipitation did not differ significantly from the capture rate during the times without precipitation (S4 File). In the other seasons, the capture rate during times with 0.5–1 mm/30 min precipitation was significantly smaller than the capture rate during times without precipitation (S4 File). The largest precipitation recorded during a time when macaques were filmed was 6.5 mm at 10:20 on August 8; however, the maximum rainfall recorded during the study period reached 54.5 mm/30 min.

## Discussion

### Seasonal patterns

Our prediction on seasonality was supported; we found that temperature positively affected the activity of Japanese macaques during the studied year, indicating that macaques were more active when the thermal conditions were favorable. The thermoneutral zone, i.e., the ambient temperature range that does not require animals to allocate additional energetic costs to thermoregulation, is reported to be 5–28°C for colobus monkeys [49], 20–27°C for titis [50], 25–30 for tarsiers [51], 25–32°C for sportive lemurs [52], above 27°C for mouse lemurs [53], 27–34°C for pygmy marmosets [54] and 17–28°C for chimpanzees [55]. Even though the thermoneutral zone for macaques has not been reported, a feeding experiment on captive macaques indicated that they changed food intake and food selection when room temperature decreased from 29 to 15°C [56]. Considering the results of that study, the reported thermoneutral zone for other species of primates, and the range of temperatures at our study site (-2.6–28.3°C), it seems that the low temperature in this habitat was unfavorable for macaques. Our results were consistent with a previous study that used behavioral observations and showed positive relations between temperature and movement activity of Japanese macaques in the same study site [25]. Other factors, such as food, may also be related to the activity patterns of Japanese macaques, but that study [25] indicated that temperature was the most important factor.

The negative effect of rainfall and its interaction with temperature also indicated that macaques were more active when conditions were favorable. We found that rainfall had a negative effect on activity and that the negative effect was more pronounced when the temperature was low. Yamane et al. [57] showed that, at an ambient temperature of 15°C, lightly clothed humans lost twice as much heat in conditions with rainfall than in conditions without rainfall, and heat production increased 2.5 times. Therefore, getting wet may impose a serious thermoregulatory problem for macaques. We hypothesize that macaques became inactive to allocate energy to thermoregulation and/or to shelter themselves from the rain in cold conditions. Accordingly, our hypothesis needs to be tested by physiological measurements of the animals’ thermal response to rainfall.

### Daily patterns

The analysis of 24-hour time period indicated that activity was high during midday and after sunrise. This partly supported our prediction, which expected macaques to be most active during the warmest time periods in a day. Comparing the activity times between transitional periods, i.e., around sunrise and around sunset, we found macaques were more active around sunrise than around sunset. This result cannot be explained from the viewpoint of thermoregulation, and thus our prediction was not supported. In our study, the temperature at the time of sunrise was 1.2°C lower than the temperature at the time of sunset. Therefore, the time around sunset must be thermally more favorable than the time around sunrise, except on very hot days. We suggest that the higher activity level seen around sunrise can be explained by the hunger of the animals after fasting overnight. By the time the macaques fall sleep around sunset, they are satiated from feeding during the day; thus, they do not need to be active. Matsuda et al. [47] reviewed daily feeding activity patterns of 19 species of non-human primates but did not report higher activity in the early morning compared to the late evening for any species. However, that same study did not show the level of activity around sunrise and sunset, and the challenging observational conditions present during the transitional times may have biased the results. Using camera traps, however, Ikeda et al. [6] showed that in Hokkaido, northern Japan, activity was higher after fasting during nighttime or daytime for diurnal Eurasian red squirrels and nocturnal raccoon dogs, respectively. Internal circadian rhythms may also play a role that affects this pattern [58]. Further studies are necessary to confirm if high activity after nighttime fasting also occurs in other animals.

When analyzing seasonal changes in daily activity patterns, the average time of filming events and the use of dawn varied with season, which was consistent with the hypothesis that macaques shift their activity to more favorable time periods based on the thermal conditions of a given season. As we predicted, during daytime, the activity of the macaques was more biased toward afternoon during the winter than during the other seasons. Moreover, their activity was more concentrated around the peak period (noon) in winter and was more dispersed in other seasons. During daytime, the temperature was highest around noon and was higher in the afternoon than in the morning. In winter, macaques shifted their time of activity to warmer, and thus more favorable, time periods. In terms of the time around dawn, when the capture rate during midday was controlled, we found a positive relationship between the activity during dawn and the daily temperature. Considering the thermoneutral zones of many species of primates [49–55], dawn during cold season must have been too cold but may not be so when the daytime temperature was relatively high. There is also the possibility that animals become active during dawn and dusk when the day length is short to compensate for the shorter available daytime. However, we can reject this possibility because we found that the capture rate at dawn decreased when the temperature was low and day length was generally short. In addition to thermoregulation, studies on cathemeral species show the effects of predation on the allocation of activity to different time periods within a day [21]. However, there is no predation pressure on the current study population [59].

Our data revealed that diurnal Japanese macaques were active during the midnight period but at a very low frequency (0.5% of the overall captures). Previous studies using video-recording equipment at the sleeping sites of this population [60, 61] enabled the detailed analysis of behavior during nighttime, but the present study revealed the quantitative differences in activity levels during daytime and nighttime. These two types of studies (recording sleeping sites and camera trapping) were complementary. In Yakushima, both video-recording at the sleeping sites [60, 61] and camera trapping (the present study) showed that Japanese macaques woke up frequently during the night, but they scarcely became active enough to leave their sleeping site and move to a different place, as they did during daytime.

The videos obtained by camera trapping were only recorded at sites where the animals were near the ground; thus, the level of ‘activity’ revealed in this study only refers to terrestrial activity. Japanese macaques may use various forest strata in response to thermal conditions or food availability [28]. Therefore, changes in capture rate may not indicate changes in overall activity; rather, they may signal a shift from terrestrial activity to arboreal activity. However, Japanese macaques do not move long distances in trees [62], so we considered that the observed decrease in the capture rate indicated an actual decrease in the level of activity, at least as far as long-distance traveling was concerned.

### Effect of rainfall

Coupled with the results obtained from using a single day as a unit of analysis, we indicated that rainfall also negatively affected the amount of activity at shorter (i.e., 30 min) time scales, so our prediction was supported. The negative effect of rainfall seemed to be less pronounced during summer because macaques did not change their activity levels during periods with the minimum amount of rainfall (0.5–1 mm/30 min) compared to periods without rainfall. In other seasons, the capture rate decreased significantly in periods with rainfall that ranged from 0.5–1 mm/30 min compared to periods without rainfall. The temperature range was calculated as 10.8–28.3°C from July to September 2014 and -2.6–23.7°C in the other seasons. Low amounts of rainfall may not impose a thermoregulatory cost on macaques on hot summer days.

Our results represented a significant step toward understanding the effect of heavy rainfall on the behavioral ecology of primates. During precipitation events greater than 6.5 mm, which constituted 2.27% of the working camera time, the number of detections of macaques was zero; in contrast, the expected number of detections that would have been made under those conditions, based on the frequency of that amount of rainfall, was 11.04. During the monsoon, or ‘tsuyu’ (i.e., rainy season), period in 2015, which was declared to last from June 2 to July 14, 2015 by the Japan Meteorological Agency, the proportion of 30-min time blocks that recorded precipitation >6.5 mm was 5.1% in our study site. On June 24, the daily rainfall reached 266 mm, and only 3.5% of time periods had no rainfall. Furthermore, 46% of the daytime had rainfall >6.5 mm on that day. On those particularly rainy days, macaques probably suffered from a serious deficiency of available activity time.

In conclusion, thermal conditions (i.e. temperature and rainfall) significantly affected the activity levels of wild Japanese macaques. Analyses of various time scales indicated that the macaques were less active under conditions with low temperature and large rainfall, and the effect of rainfall was less pronounced when the temperature was high. However, the daily activity pattern was not explained solely by thermal condition; in fact, macaques were more active around sunrise than around sunset, even though it was colder around sunrise. This pattern may be related to overnight fasting. We should note that the present study was conducted in a cool-temperate forest, where cold stress was likely to be particularly significant to the animals. Given that animals may exhibit different behaviors and activity patterns in response to climatic variations, we suggest that further studies on the effects of climate are necessary and should be carried out in various habitats, such as tropical rain forest or savanna, to better understand the effects of thermal conditions on the activity levels of animals.

## Supporting information

S1 File Supporting information 1. Data on the working period of the cameras.
(XLSX)

S2 File Supporting information 2. Data on the time of the filming events of Japanese macaques and the sunrise and sunset times for each event.
(XLSX)

S3 File Supporting information 3. Data on the rainfall during the study period.
(XLSX)

S4 File Supporting information 4. Results of the statistical analysis.
(XLSX)
